# Effects on atrial fibrillation symptom burden of an obesity-focused cardiac rehabilitation program: Insights from the OPTICARE XL trial

**DOI:** 10.1007/s12471-026-02079-x

**Published:** 2026-09-09

**Authors:** Nicole Tenbült-van Limpt, Richard A. J. Post, Nienke ter Hoeve, Iris den Uijl, Madoka Sunamura, Yuan Lu, Eric Boersma, Rita van den Berg-Emons, Hareld Kemps, Rutger Brouwers

**Affiliations:** 1 Department of Cardiology, Máxima Medical Centre, Eindhoven/Veldhoven, The Netherlands; 2 Department of Industrial Design, Eindhoven University of Technology, Eindhoven, The Netherlands; 3 Department of Biostatistics, Erasmus University Medical Center, Rotterdam, The Netherlands; 4 Department of Epidemiology, Erasmus University Medical Center, Rotterdam, The Netherlands; 5 Department of Rehabilitation Medicine, Erasmus University Medical Center, Rotterdam, The Netherlands; 6 Capri Cardiac Rehabilitation, Rotterdam, The Netherlands; 7 Department of Cardiology, Thoraxcenter, Erasmus University Medical Center, Rotterdam, The Netherlands

**Keywords:** Atrial fibrillation, Obesity, Cardiac rehabilitation

## Introduction

Obesity is a major modifiable risk factor for atrial fibrillation (AF) [1]. Sustained weight loss and regular exercise are therefore key components of AF management [2]. In symptomatic AF populations, comprehensive risk factor management programmes targeting weight loss, physical activity, and behavioural change have been associated with reduced AF recurrence and symptom burden [1, 3, 4]. Cardiac rehabilitation (CR) provides a scalable framework for lifestyle modification, risk factor management (including weight loss), and improvement of exercise capacity and quality of life [5]. Whether CR, particularly when tailored for patients with obesity, is effective to reduce AF symptom burden remains uncertain [6]. We therefore evaluated the effect of an obesity-focused CR programme on AF symptom burden in patients with non-valvular AF [7].

Data from the OPTImal CArdiac REhabilitation XL (OPTICARE XL) trial were used. This was an open-label randomized controlled trial conducted between February 2017 and January 2019 in the Netherlands. Adults with obesity (body mass index [BMI] ≥ 30 kg/m^2^) referred for CR because of coronary artery disease or documented non-valvular AF were eligible. The study was approved by the local ethics committee and prospectively registered (MEC-2016-622, NL5589).

Participants were randomized to either a 1-year obesity-focused CR programme (OPTICARE XL) or standard CR. OPTICARE XL consisted of 12 weeks of supervised group-based aerobic and resistance training twice weekly, using obesity-tailored exercise modalities delivered in small groups combined with structured behavioural coaching targeting weight reduction, disease coping and active lifestyle supported by an activity tracker, followed by a 9-month aftercare phase with booster sessions. Standard centre-based CR was delivered according to ESC guidelines [8], also group-based, and included supervised exercise training (6–12 weeks) and lifestyle education. Beyond differences in exercise modality, group composition, and programme duration, OPTICARE XL incorporated peer-group interaction and behavioural change techniques tailored to obesity, aimed at enhancing self-monitoring, relapse prevention, and coping with chronic disease. Outcome measures were assessed at baseline, 3 months after the start of CR, and 6 months after completion of the CR programme (i.e. approximately 12 months after baseline). Of 201 patients enrolled in the parent trial, 68 with documented non-valvular AF were included in this exploratory post hoc subgroup analysis (OPTICARE XL, *n* = 40; standard CR, *n* = 28) derived from the OPTICARE XL trial [7].

The primary outcome of the current paper was AF symptom burden at 18 months, assessed using the University of Toronto Atrial Fibrillation Severity Scale (AFSS; total score range 3–30, higher scores indicating greater symptom burden). The AFSS reflects patient-reported symptom frequency, duration, and severity. The primary outcome was assessed 18 months after baseline, corresponding to approximately 6 months after CR completion. Secondary outcomes included BMI, AF symptom number, frequency and severity (AFSS domains). Analyses followed the intention-to-treat principle. We used linear mixed-effects models with patient as a random effect and fixed effects for treatment, time, and their interaction. Missing outcome data were handled by linear mixed-effects models under a missing-at-random assumption. Statistical inference was based on 95% confidence intervals.

Baseline characteristics are presented in Tab. 1. The treatment groups were generally comparable at baseline.

**Table 1:** Baseline characteristics of participants

*Characteristic*	*OPTICARE XL CR (n=40)*	*Standard CR (n=28)*
Male sex, *n* (%)	22 (55)	19 (68)
Age, years	62.2 ± 10.1	62.8 ± 10.9
BMI, kg/m^2^	36.4 ± 4.3	35.7 ± 4.5
Previous PVI, *n* (%)	7 (18)	4 (14)
AFSS total score (range 3–30)	14.6 ± 4.4	16.4 ± 6.5
AF symptom number (range 0–16)	9.2 ± 4.2	9.7 ± 3.5
AF symptom frequency (range 16–80)	31.5 ± 8.4	32.2 ± 7.9
AF symptom severity† (range 0–48)	0 (8)	6.5 (18)

† Data are presented as mean ± SD or *n* (%). AF symptom severity is presented as median (IQR)

Abbreviations: *AF* atrial fibrillation, *BMI* body mass index, *CR* cardiac rehabilitation, *PVI* pulmonary vein isolation

**Fig. 1:**
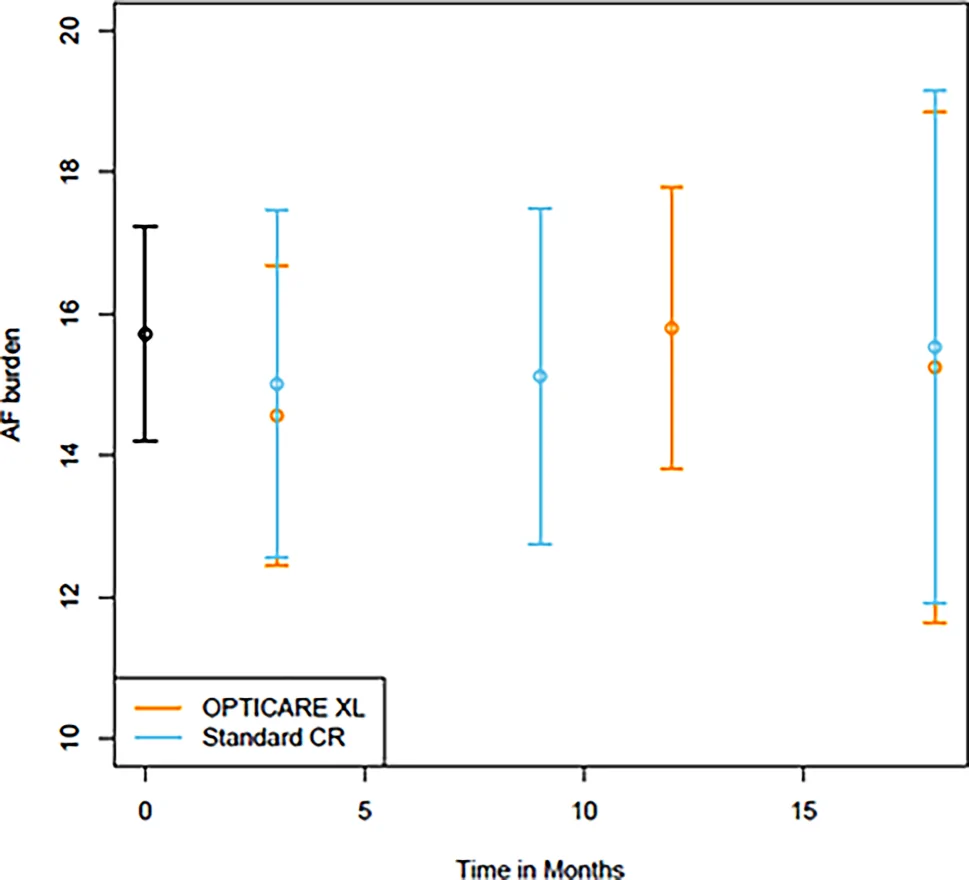
Model-estimated mean AF symptom burden (AFSS total score) over time with 95% confidence intervals in patients randomized to OPTICARE XL or standard cardiac rehabilitation. Estimates were derived from linear mixed-effects models adjusting for time, treatment, and their interaction. Baseline values represent observed means, whereas follow-up values represent model-estimated marginal means

At 18 months, AFSS scores showed no significant change from baseline in either the standard CR group (mean 15.5; mean difference −0.2) or the OPTICARE XL group (mean 15.2; mean difference −0.5). There were no significant differences in AFSS scores between OPTICARE XL and standard CR at 3 months (−0.44, 95% CI: −3.29 to 2.40), 12 months (0.68, 95% CI: −1.84 to 3.20), or 18 months (−0.28, 95% CI: −4.52 to 3.95) (Fig. 1). AF symptom number decreased significantly after 18 months in the standard CR group (mean difference −3.55, 95% CI: −5.46 to −1.63), whereas no significant change was observed in the OPTICARE XL group (mean difference −1.13, 95% CI: −2.89 to 0.61). No significant between-group differences were observed. AF symptom frequency decreased significantly after 18 months in the standard CR group (mean difference −4.42, 95% CI: −8.30 to −0.54), whereas no significant change was observed in the OPTICARE XL group (mean difference −2.90, 95% CI: −6.23 to 0.42). No significant between-group differences in AF symptom frequency were found. AF symptom severity did not change significantly over time in either group.

At 18 months, BMI decreased by 0.93 kg/m^2^ in the standard CR group (95% CI: −2.03 to 0.17) and by 0.99 kg/m^2^ in the OPTICARE XL group (95% CI: −1.68 to −0.31). However, between-group differences in BMI change were not significant.

In this AF subgroup analysis of the OPTICARE XL trial, neither standard CR nor an obesity-focused CR programme reduced AF symptom burden over 18 months.

The modest weight loss achieved in the XL group did not translate into meaningful symptom improvements. This contrasts with findings from the recent POP-AF trial, which demonstrated improved AF-related outcomes following an intensive lifestyle intervention [9]. A possible explanation for the absence of treatment effect is that the OPTICARE XL intervention relied on scheduled consultations and reinforcement sessions, which probably offered insufficient opportunity for continuous lifestyle and symptom monitoring or timely, personalised feedback, increasing relapse risk. Future research should evaluate interventions that provide more sustained guidance and timely feedback to support long-term behavioural change and improve AF-related outcomes. Future interventions should link personalised lifestyle guidance to key AF triggers (e.g. exercise intensity, sleep disruption, alcohol intake), enabling dynamic adjustment of behavioural targets. Such guidance could be delivered through hybrid interventions incorporating prolonged monitoring of lifestyle behaviour and data-enabled real-time feedback, supporting more responsive and personalised adjustments in exercise, diet, and behavioural guidance. This approach is supported by contemporary cardiac telerehabilitation frameworks highlighting continuous monitoring and timely feedback [10].

## Data Availability

All data supporting the findings of this work are included in the article and its supplementary materials, where applicable.
